# Endocrine and metabolic effects of GLP-1 receptor agonists on women with PCOS, a narrative review

**DOI:** 10.1530/EC-24-0529

**Published:** 2025-03-21

**Authors:** Marine Monney, Maria Mavromati, Sophie Leboulleux, Karim Gariani

**Affiliations:** ^1^ Division of Endocrinology, Diabetes, Nutrition and Therapeutic Patient Education, Department of Medical Specialties, Geneva University Hospitals, Geneva, Switzerland; ^2^ Faculty of Medicine, University of Geneva, Geneva, Switzerland; ^3^ Diabetes Center, Faculty of Medicine, University of Geneva, Geneva, Switzerland

**Keywords:** polycytic ovary syndrome, glucagon-like peptide-1, metabolic disorder, obesity

## Abstract

Polycystic ovary syndrome (PCOS) is a common endocrine disorder in women of reproductive age. This condition is associated with various hormonal, reproductive and metabolic alterations, including androgen excess, ovulatory disorders and a hyperinsulinemic state. A personalized therapeutic approach is necessary to improve PCOS, focusing on patients’ main concerns, with the goal of addressing ovarian dysfunction, reducing hyperandrogenism and improving metabolic alterations, particularly through weight reduction. The therapeutic class of glucagon-like peptide-1 receptor analogues (GLP-1 RAs) represents an attractive option for PCOS due to its various beneficial effects, such as weight loss. In this review, we discuss the clinical and pathological aspects of PCOS, as well as the data and potential roles of GLP-1 RAs in managing this condition.

## Introduction

Polycystic ovary syndrome (PCOS) is the most prevalent endocrine disorder among women of reproductive age. This heterogeneous condition is characterized by hyperandrogenism, ovarian dysfunction and metabolic issues such as insulin resistance, visceral adiposity and obesity (1). PCOS significantly impacts health and quality of life, highlighting the importance of timely diagnosis and appropriate management (2).

The pathogenesis of PCOS is complex, involving interactions between genetic predisposition and environmental factors. Management strategies require a multidisciplinary, personalized approach to address each patient’s symptoms. Key objectives include restoring ovulation, reducing androgen levels and supporting conception. Improving metabolic complications, particularly through weight loss, is also crucial. GLP-1 receptor analogues (GLP-1 RAs) offer a promising therapeutic avenue, as they facilitate weight reduction, improve metabolic markers such as insulin sensitivity and lipid profiles, and also reduce the incidence of T2DM, which are more prevalent in women with PCOS. In addition, GLP-1 RA therapies may lower cardiovascular risks, which are elevated in PCOS, and potentially enhance ovulatory function through direct ovarian effects or weight-related improvements (3). This review examines PCOS pathogenesis and the potential roles of GLP-1 RAs based on current evidence.

### PCOS

PCOS affects approximately 10% of women of reproductive age (1), presenting with ovulatory dysfunction, androgen excess (clinical or biochemical) and polycystic ovaries. Its manifestations are diverse, encompassing fertility challenges, psychological distress and metabolic complications with lifelong implications (4). The 2023 International Evidence-based Guideline introduced streamlined diagnostic criteria, including anti-Müllerian hormone (AMH) as an alternative to ovarian ultrasound for adults, while adolescents must meet criteria for oligo/anovulation and androgen excess (5). These guidelines also emphasize significant metabolic risks, such as insulin resistance, T2DM, sleep apnea, cardiovascular disease and adverse pregnancy outcomes, as well as its profound impact on quality of life (6, 7).

Despite its prevalence, PCOS often remains underdiagnosed and inadequately managed, with delays stemming from limited awareness and insufficient physician education. Management strategies focus on symptomatic treatments, including androgen excess, regulating ovulation (via contraception, fertility counseling or induction as needed) and preventing endometrial hyperplasia or carcinoma. Attention is also given to metabolic health, along with psychological issues such as depression and anxiety.

Women with PCOS face a higher prevalence of impaired glucose tolerance and type 2 diabetes (8), regardless of age or body mass index (BMI). Cardiovascular risk, although generally low in premenopausal women, is elevated in this population (9). Metabolic syndrome affects 33–47% of women with PCOS, compared to 18–19% in the general premenopausal population. Insulin resistance is a common feature, observed in 75% of women with a BMI below 27 kg/m^2^ and up to 95% in those with a BMI over 30 kg/m (2, 10, 11, 12, 13, 14). Furthermore, the relative risk of type 2 diabetes is tripled in women with PCOS compared to matched controls (15).

Among women with PCOS, there is a subgroup known as lean PCOS, which accounts for approximately one-quarter of all PCOS cases (16). This subgroup is characterized by a BMI of less than 25 kg/m^2^ and may present with hyperandrogenism and irregular or absent menstrual cycles but with a less severe metabolic impact compared to women with PCOS and obesity (17). In this specific form of PCOS, weight loss is not recommended, and therefore lifestyle interventions, pharmacological treatments such as GLP-1 RAs, or even surgical approaches such as bariatric surgery are not considered appropriate (16).

### Pathogenesis of PCOS, adipose tissue dysfunction and insulin resistance

The ovary in PCOS is characterized by steroidogenic hyperactivity with excess androgen production from theca cells, as well as hypersensitivity to luteinizing hormone (LH) (2). There is also granulosa cell dysfunction with impaired folliculogenesis, which seems to further worsen thecal androgen production (18). Functional adrenal hyperandrogenism is also found in 25% of PCOS cases, but its pathogenesis remains unclear. Finally, about 10% of PCOS cases show no evidence of functional hyperandrogenism (either ovarian or adrenal) and are milder cases, most of them attributed to obesity (2).

Four different PCOS phenotypes have been described (2). Phenotype 1 includes all three features of PCOS, androgen excess, ovulatory dysfunction and polycystic ovaries, and is considered as being the classic mode of presentation of the syndrome, while phenotype 2 combines only ovulation dysfunction and androgen excess. Phenotype 3 only includes hyperandrogenism and polycystic ovarian morphology without oligo/anovulation, whereas phenotype 4 which combines oligo/anovulation and polycystic ovaries but no androgen excess, is less specific and thus controversial. It is interesting to state that as the severity of androgen excess decreases from phenotype 1 to 4, the severity of insulin resistance also seems to decrease.

The exact mechanism of insulin resistance in PCOS, regardless of BMI, has not yet been fully understood and described. Insulin resistance leads to increased lipolysis and gluconeogenesis. Compensatory hyperinsulinemia induces overstimulation of non-insulin-sensitive tissues, including ovary (19). Insulin resistance in PCOS increases androgen production from ovarian theca cells both directly and by increasing sensitivity to LH stimulation (20). Insulin also increases the expression of insulin receptors, LH receptors and insulin-like growth factor (IGF) receptors on granulosa cells, thus impairing folliculogenesis. Folliculogenesis is further impaired by hyperinsulinemia through the dysregulation of the hypothalamic–pituitary–ovarian axis (19). In addition, hyperinsulinemia decreases the production of sex hormone-binding globulin (SHBG) from the liver and increases the sensitivity of adrenal steroidogenesis to adrenocorticotropic hormone (ACTH) stimulation, both of them contributing to hyperandrogenism (2, 21). Despite the fact that insulin resistance seems to be intrinsic to PCOS, weight excess and obesity further worsen the situation.

White adipose tissue exhibits several alterations in women with PCOS, contributing to the pathophysiology of this disorder. Among these modifications is adipocyte hypertrophy, as demonstrated by studies showing that women with PCOS have a larger adipocyte volume compared to women without PCOS, matched for BMI (22, 23). The mechanism underlying the induction of adipocyte cellular dysfunction by hypertrophy is not fully established, but one possibility is that a cellular hypoxia phenomenon is induced by hypertrophy, particularly related to insufficient vascular perfusion in proportion to cell enlargement (24). The distribution of white adipose tissue also appears to be altered in women with PCOS, with a tendency toward a higher amount of visceral adipose tissue (VAT), knowing that VAT is metabolically more active and contributes to insulin resistance and hyperandrogenism (25). Hyperplasia plays a key role in adipocyte physiology by limiting hypertrophy and preventing cellular dysfunction. In PCOS, however, adipogenesis seems to be disrupted. Studies have shown that exposure of adipocyte cells from surgical biopsies to androgens interferes with the progression of stem cells into pre-adipocytes, as well as the differentiation of pre-adipocytes into mature adipocytes (26, 27). In adipose tissue, the insulin resistance observed in PCOS is thought to be associated with a reduction in both the content and expression levels of GLUT-4, a key transporter involved in glucose uptake. This decrease in GLUT-4 expression impairs the ability of insulin to facilitate glucose entry into adipocytes, contributing to the overall insulin resistance observed in women with PCOS (28).

Hyperandrogenism, a hallmark of PCOS, is not only driven by ovarian theca cells but also involves other tissues, including white adipose tissue and adrenal cortex cells, which contribute to the excessive production of androgen hormones in this condition (29, 30). In adipose tissue in PCOS, increased intra-adipose concentrations of testosterone have been identified. Mechanistically, the processes contributing to excessive androgen production in white adipose tissue include the ability of human adipose tissue to actively synthesize androgens, catalyzed by 17β-HSD5, and the increased expression of the androgen-activating enzyme aldo-ketoreductase type 1 C3 (AKR1C3) (31, 32).

### Glucagon-like peptide-1 (GLP-1) receptor agonists

GLP-1 is a hormone that has been shown to have numerous positive metabolic effects, especially in the treatment of diabetes. These effects include stimulating insulin secretion and inhibiting glucagon in the pancreas, promoting a sense of satiety via the hypothalamus, and slowing gastric emptying in the digestive tract. Endogenously, GLP-1 is a gut hormone produced by intestinal L cells, primarily located in the ileum, in response to food intake (33). Due to rapid degradation by the enzyme dipeptidyl peptidase-4 (DPP-4) within minutes, the pharmacological use of native GLP-1 was not feasible. To address this, GLP-1 RAs were developed, which are resistant to degradation by DPP-4 and capable of mimicking GLP-1’s action. Various GLP-1 RAs have since been introduced to the market, with progressively longer half-lives, reducing injection frequency from twice daily for older formulations to once daily and finally to once weekly.

This therapeutic class was quickly recognized for its significant potential in weight management due to its ability to induce a feeling of gastric fullness and regulate food intake. As a result, it is now used not only for diabetes management but also in cases of obesity without associated diabetes. In addition to glycemic control and weight loss, GLP-1 receptor agonists have demonstrated further benefits, with recent studies highlighting their cardiovascular and renal protective effects (34). A growing body of evidence suggests that this therapeutic class could also have positive effects in other conditions, such as cancer and dementia (35, 36). GLP-1 receptors are expressed in various tissues, including the hypothalamus, pancreas, smooth muscle, adipose tissue, kidneys, heart and lungs (37). In addition, some evidence suggests that GLP-1 receptors may also be expressed in the ovaries. One study showed GLP-1 receptor expression in human ovarian tumor cells, while another found their presence in the membrane and cytoplasm of mouse ovarian granulosa cells (38, 39). This provides a rationale for the potential role of GLP-1 receptor agonists in the pathogenesis of PCOS. In addition, their broad expression in other tissues may have indirect effects, particularly on ovarian tissue.

Of note, some observational studies had suggested a potential link between the use of GLP-1 RAs and the occurrence of psychiatric disorders such as severe depression. However, recent research has not confirmed this association. Notably, a retrospective registry study involving over 300,000 patients in Sweden and Denmark found no increased risk of depression or anxiety diagnoses within the first year of initiating GLP-1 RA therapy (40). In addition, a randomized study of nearly 4,000 individuals followed for 68 weeks found no association between semaglutide use and the development of severe depression or suicidal ideation. These findings are reassuring and suggest that GLP-1 RAs do not pose a significant risk for severe psychiatric disorders (41).

### Benefits of GLP-1 RAs in women with PCOS

Weight loss is a cornerstone in the therapeutic management of many cases of PCOS (5, 42). The recommended approach to achieving weight loss is multimodal, combining dietary interventions, regular physical activity and behavioral therapy, with additional options such as pharmacotherapy and bariatric surgery for patients requiring more intensive or specialized interventions (43).

In women with PCOS and weight excess, weight loss can lead to improvements in various hormonal, cardiometabolic and reproductive parameters. Benefits include reductions in hyperandrogenism, insulin resistance and cardiovascular risk, as well as the restoration of regular menstrual cycles. However, achieving a significant weight reduction of at least 5% is often challenging with lifestyle interventions alone for the majority of women with PCOS (44). In this context, GLP-1 RAs represent an attractive therapeutic approach, addressing several aspects of PCOS pathophysiology. GLP-1 RAs can partially counteract insulin resistance, a common issue in PCOS. This therapeutic class has been shown in numerous studies to reduce insulin resistance not only through weight reduction but also by lowering inflammation, reducing oxidative stress, modulating lipid metabolism homeostasis and increasing glucose transporter activity (45). This approach is expected to improve metabolic parameters, potentially reduce cardiovascular events and address reproductive function disorders. The hypothalamus–pituitary–gonadal (HPG) axis represents another potential therapeutic target for GLP-1 RAs in PCOS. *In vitro* studies using a murine hypothalamic cell line have shown that the GLP-1 RA exendin-4 stimulates GnRH (gonadotropin-releasing hormone) release in a dose-dependent manner. In addition, this study found that intracerebroventricular administration of exendin-4 in male rats led to a rapid increase in LH levels (46). An *in vivo* study on female rats demonstrated that administration of GLP-1 RAs increased the amplitude of LH secretion during the pre-ovulation phase (47). This effect was associated with higher levels of estradiol and progesterone and an increase in the number of Graafian follicles during the estrous cycle. Mechanistically, the positive impact of GLP-1 on the HPG axis appears to be mediated through the activation of the hypothalamic kisspeptin system, a major regulator of GnRH release (47).

It remains to be determined whether GLP-1 RAs reduce androgen production in white adipose tissue through a direct action or via an indirect mechanism linked to the reduction in white adipose tissue mass, which may be associated with decreased food intake induced by this therapeutic class. The reduction in androgen secretion observed with GLP-1 RAs is believed to result primarily from systemic secondary mechanisms, such as metabolic improvements or changes in adipose tissue dynamics. However, a potential direct effect on ovarian tissue or adrenal steroidogenesis is possible and warrants further investigation.

Altogether, it appears that GLP-1 RAs can positively impact several pathological aspects of PCOS, including insulin resistance, dysregulation of GnRH release and maturation disorders of ovarian follicles. This suggests that GLP-1 RAs represent a promising therapeutic opportunity.

### Bariatric surgery and PCOS

Bariatric surgery has recently gained attention as a promising treatment option for managing PCOS. The most common types of bariatric procedures include Roux-en-Y gastric bypass, vertical sleeve gastrectomy and gastric banding (BAND). However, obesity play a critical role in the pathophysiology of PCOS, only a few studies have explored the effectiveness of bariatric surgery as a treatment for women with this condition, and most of the available evidence stems from prospective cohort studies. Various hormonal changes occur after bariatric surgery, including an increase in the levels of the anorexigenic hormones GLP-1 and PYY, and a reduction in ghrelin levels, an orexigenic hormone involved in stimulating appetite (48).

These hormonal changes are potentially beneficial in the context of PCOS due to their positive effects on appetite regulation, metabolism and the hormonal imbalances associated with the condition. Prospective studies have highlighted the benefits of bariatric surgery in women with PCOS, including metabolic improvements such as significant weight loss and enhanced glucose homeostasis, as well as hormonal improvements, notably with significant reductions in total testosterone levels.

A recent multicenter randomized trial, the BAMBINI trial, assessed the effectiveness of bariatric surgery compared to medical treatments in promoting spontaneous ovulation in women with PCOS, obesity and either oligomenorrhea or amenorrhea. The study enrolled a total of 80 women and demonstrated superior ovulation rates in the bariatric surgery group, suggesting a potential improvement in spontaneous fertility (49).

It remains to be determined whether these benefits are solely mediated by weight loss or also by hormonal changes following bariatric surgery, such as the increase in GLP-1 levels. Furthermore, within a personalized medicine approach, it will be crucial to determine which option offers greater benefit for an obese woman with PCOS: treatment with GLP-1 RAs or bariatric surgery.

## Effects of GLP-1 RAs on PCOS in preclinical studies

Several preclinical and clinical studies have evaluated this treatment across various pathological aspects of PCOS, including metabolic disorders with insulin resistance, hormonal and/or clinical hyperandrogenism and ovulatory dysfunction. In this review, we will explore various animal studies and human clinical trials.

There are several animal models of PCOS, including prenatal, peripubertal, postmenopausal exposure and genetic models. The dehydroepiandrosterone (DHEA)-induced PCOS model, with or without the addition of an obesogenic diet, is likely the most widely used. It replicates several key aspects of PCOS, including elevated testosterone and LH levels, irregular cycles, ovarian cysts and metabolic disturbances such as adipose tissue dysfunction and obesity. In addition, on a psychological level, it tends to induce anxiety. Various genetic models of PCOS have also been developed, such as Erbb-4-knockout mice, Dendd1a-knockin and knockout mice, and Cyp17 theca cell (TC17) overexpression mice (50).

The effectiveness of GLP-1 RAs on preclinical models of PCOS has primarily been tested using dihydrotestosterone (DHT)-induced PCOS models. In studies involving DHEA-induced PCOS models, the addition of liraglutide or semaglutide was associated with reductions in insulin and androgen levels, as well as inflammation (51). Mice treated with GLP-1 RAs exhibited lower levels of testosterone and LH, as well as improvements in ovarian morphology. In addition, another study using a similar PCOS model and treatment approach with liraglutide or semaglutide demonstrated not only metabolic and hormonal improvements but also a restoration of the composition and diversity of the intestinal microbiota (52).

In another study using a comparable model with female Sprague Dawley rats treated with DHT, the addition of liraglutide led to improvements in several metabolic factors, including reduced weight and insulin resistance, increased leptin levels and an improved lipid profile (53). In addition, research combining *in vitro* and *in vivo* approaches suggests that GLP-1 may have anti-apoptotic and regulatory effects on mural granulosa cells (MGCs), which are involved in steroid hormone secretion and play a crucial role in oocyte development. Mechanistically, the action of GLP-1 appears to be at least partially mediated through the modification of phosphorylation sites on forkhead box protein O1 (38).

These observations suggest that GLP-1 could contribute to the viability of MGCs and support oocyte maturation, potentially aiding in the regulation of menstrual cycles. There is preclinical evidence indicating that GLP-1 RAs may counteract the pathogenesis of PCOS by improving various metabolic and reproductive parameters. However, the exact mechanisms remain to be fully elucidated, and several hypotheses have been proposed.

## Effects of GLP-1 RAs on PCOS in clinical studies

### Liraglutide in PCOS

Numerous studies have been conducted on the use of liraglutide for weight loss in women with PCOS, their characteristics and results are summarized in Table 1. In women newly diagnosed with PCOS, liraglutide monotherapy led to significant reductions in weight, BMI and waist circumference (WC), but comparable results were found in the metformin group (54). Among women with PCOS who did not achieve a 5% body weight reduction during 6 months of treatment with metformin, Jensterle *et al.* showed a significant improvement in eating behavior and weight loss following a switch to liraglutide (55). Since metformin’s effect on weight reduction is often unsatisfactory, they also investigated the potential add-on effect of treatment with liraglutide on weight loss for non-diabetic women with PCOS. In addition to lifestyle modification, using a combination of metformin and liraglutide led to more significant weight loss, a decrease in BMI and WC than metformin alone (56, 57). The authors concluded that metformin may have an additional impact on the weight-lowering ability of GLP-1 RAs by enhancing the incretin axis after GLP-1 stimulation. This enhancement leads to increased expression of the GLP-1 receptor and the insulinotropic receptor. To assess these hypotheses, they conducted a short-term study comparing the weight-lowering potential of liraglutide 1.2 mg in combination with metformin to liraglutide monotherapy at a higher dose (3 mg) in women with PCOS (58). Both treatment groups showed a significant improvement in obesity-related measures (high dose of liraglutide showing superiority over the combination therapy). However, the combination therapy displayed better tolerability and a more positive impact on androgen levels, suggesting that a dual treatment approach may be more beneficial for managing PCOS compared to a single high-dose medication. Recently, Elkind-Hirsh *et al.* showed that treating non-diabetic women with obesity and PCOS with liraglutide 3 mg a day for 32 weeks not only led to weight loss but also improved hyperandrogenism (free androgen index – FAI) and menstrual cycle frequency compared to a placebo (59).

**Table 1:** Metabolic and endocrine outcomes of liraglutide in PCOS.

Reference	Trial characteristics	Metabolic characteristics	Intervention group	Comparative group	Metabolic outcomes	Endocrine outcomes
Jensterle *et al.* 2014 (47)	Single center, randomized, prospective, open label, 12 weeks, with 40 participants	Women with PCOS	Liraglutide 1.2 mg a day sc plus metformin 1,000 mg BID	Liraglutide 1.2 mg a day sc	• **Body weight, BMI, WC significantly decreased** • HOMA-IR decreased (no difference between groups)	• Menstrual frequency no significant change
				Metformin 1,000 mg BID		
Jensterle *et al.* 2015 (44)	Single center, randomized, prospective, 12 weeks, with 32 participants	Women with new PCOS	Liraglutide 1.2 mg a day sc	Metformin 1,000 mg BID	**•** **Body weight, BMI, WC significantly decreased (no difference between group)** • HOMA-IR not changed	• Menstrual frequency no significant change
Jensterle *et al.* 2016 (46)	Single center, randomized, prospective, open-label, 12 weeks, with 44 participants	Women with POCS	Liraglutide 1.2 mg a day sc plus metformin 1,000 mg BID	Metformin 1,000 mg BID	• **Body weight, BMI, WC significantly decreased (no difference between group** • FPG, OGTT 2 h glucose and HOMA-IR significantly decreased (no difference)	• Total and free testosterone reduced and SHBG raised (no difference between groups)
Jensterle *et al.* 2017 (48)	Single center, randomized, prospective, open-label, 12 weeks, with 30 participants	Women with PCOS	Liraglutide 3 mg a day	Liraglutide 1.2 mg a day plus metformin 1,000 mg BID	**•** **Body weight, BMI, WC significantly decreased (difference between group for BMI and waist)** • HOMA-IR decreased (no difference between groups)	• Total and free testosterone reduced and SHBG raised (no difference between group)
Jensterle *et al.* 2015 (50)	Single center, randomized, prospective, open-label, 12 weeks, with 45 participants	Women with PCOS	Liraglutide 1.2 mg a day sc	Metformin 1,000 mg BID	• **Body weight significantly decreased in liraglutide and roflumilast (no difference)** • HOMA-IR decreased (not difference between group)	• No effect on total and free testosterone, SHBG, androstenedione, DHEAS, LH, FSH • Menstrual frequency increased (not significantly, no difference between group)
				Roflumilast 500 μg a day		
Elkind-Hirsch *et al.* 2022 (49)	Single center, randomized, double blind, placebo-controlled, 32 weeks, with 82 participants	Women with PCOS	Liraglutide 3 mg a day	Placebo	• **Bodyweight, BMI, WC significantly decreased** • FPG, OGTT, HOMA-IR significantly improved	• **FAI significantly decreased** • Menstrual frequency significantly increased
Nylander *et al.* 2017 (51)	Single center, randomized, double blind, placebo-controlled, 26 weeks, with 72 participants	Women with PCOS	Liraglutide up to 1.8 mg a day sc	Placebo	• Bodyweight significantly decreased	• **Menstrual frequency (bleeding ratio) significantly increased** **•** **Ovarian volume decreased •** **AMH, LH, FSH, estradiol not changed** **•** **Free testosterone, FAI significantly decreased** **•** **SHBG significantly increased**
Xing *et al.* 2022 (52)	Single center, randomized, open-label, parallel-group, controlled study, 12 weeks, with 60 participants	Women with PCOS	Liraglutide 1.2 mg a day sc plus metformin 1,000 mg BID	Metformin 1,000 mg BID	• Body weight, BMI, WC significantly decreased (no difference between groups) • FPG, HOMA-IR significantly decreased (no difference between groups)	**•** **Significant menstrual recovery (no difference between groups)** **•** **Estradiol, SHBG increased (no difference between groups)** **•** **LH, total testosterone and FAI significantly decreased; progesterone significantly increased**
Salamun *et al.* 2018 (53)	Single center, randomized, prospective, open-label, 12 weeks, with 28 participants	Infertile women with PCOS	Liraglutide 1.2 mg a day sc plus metformin 1,000 mg BID	Metformin 1,000 mg BID	• Body weight, BMI, WC significantly decreased (no difference between groups) • FPG and post OGTT, HOMA-IR decreased significantly	• SHBG significantly increased • **Pregnancy rate per embryo transfer significantly higher (85.2% vs 28.6%)**

Primary outcomes of the trials are shown in bold. Significant differences are specified in the table. ‘No difference between groups’ is stated when effects are seen in both groups but not significantly different.

Abbreviations: PCOS, polycystic ovary syndrome; sc, subcutaneous; BID, bis in die; BMI, body mass index; HOMA-IR, homeostatic model assessment for insulin resistance; FPG, fasting plasma glucose; OGTT, oral glucose tolerance test; SHBG, sex hormone-binding globulin; DHEAS , dehydroepiandrosterone sulfate; LH, luteinizing hormone; FSH, follicle-stimulating hormone; FAI, free androgen index; AMH, anti-Müllerian hormone; WC, waist circumference.

Jensterle *et al.* also aimed to evaluate the effect of roflumilast on body weight compared to metformin and liraglutide. Roflumilast is a selective inhibitor of phosphodiesterase enzyme 4 (PDE4) involved in GLP-1 release. Short-term (12 weeks) treatment with a PDE4 inhibitor led to significant weight loss in women with PCOS. The same result was seen with liraglutide but not with metformin (60). Considering roflumilast as a potential new treatment for women with PCOS requires further trials due to the small sample size, short duration of the study, and lack of other comparative studies.

The decrease in obesity-related features through GLP-1 treatment was linked to positive effects on fasting glucose, insulin sensitivity (homeostatic model assessment for insulin resistance – HOMA-IR), metabolic syndrome and hormonal markers (testosterone, androstenedione and SHBG), but some studies were unable to demonstrate clinical endocrine benefits (menstrual frequency) (54, 56, 57). Most of them have not been designed to assess endocrine effects, and the results emerged from secondary outcomes. Nylander *et al.* aimed to investigate the impact of liraglutide on various indicators of ovarian dysfunction (such as bleeding ratio, ovarian morphology, levels of AMH and androgens) in a 26-week double-blind, placebo-controlled randomized trial involving women with PCOS (61). Improvement in bleeding regularity, a decrease in ovarian volume and free testosterone levels, and an increase in SHBG levels were reached with liraglutide treatment, whereas no effect on AMH, LH, follicle-stimulating hormone (FSH) levels and Ferriman–Gallwey score was observed in either group. Ninety-two percent of women with PCOS achieved menstrual cycle recovery (defined as the return of regular menstrual cycles) and showed a significant improvement in biological markers (such as LH, FSH, estradiol, progesterone, total testosterone, SHBG and FAI) when they received combination therapy with liraglutide and metformin (62). The potential impact of liraglutide on fertility has been assessed in 28 infertile women with PCOS (63). A short-term preconception intervention with low-dose liraglutide added to metformin improved the *in vitro* fertilization pregnancy rate per embryo transfer and cumulative pregnancy rate compared to metformin alone, even though the weight reduction was similar.

### Exenatide in PCOS

Eight studies have examined the metabolic and/or endocrine effects of administering exenatide to women with PCOS. Table 2 summarizes their characteristics and results.

**Table 2:** Metabolic and endocrine outcomes of exenatide in PCOS.

Reference	Trial characteristics	Metabolic characteristics	Intervention group	Comparative group	Metabolic outcomes	Endocrine outcomes
Elkind-Hirsch *et al.* 2008 (58)	Single center, randomized, prospective, open label, 24 weeks, with 60 participants	Overweight women with PCOS	Exenatide 10 μg BID plus metformin 1,000 mg BID	Exenatide 10 μg BID	• Body weight and BMI significantly decreased • HOMA-IR decreased (no difference between groups)	• **Menstrual frequency significantly better** • Ovulation rate significantly higher • Testosterone decreased significantly, SHBG raised (no difference between groups)
				Metformin 1,000 mg BID		
Ma *et al.* 2021 (57)	Single center, randomized, prospective, open label, 12 weeks, with 50 participants	Women with PCOS	Exenatide 2 mg QW plus metformin 500 mg TID	Metformin 500 mg TID	**•** **Body weight, BMI, WC significantly decreased** • FPG, OGTT 2 h glucose and insulin significantly decreased • HOMA-IR not changed	• Testosterone significantly decreased (no difference between groups)
Zheng *et al.* 2017 (56)	Single center, randomized, prospective, open label, 12 weeks, with 82 participants	Women with PCOS	Exenatide 10 μg BID	Metformin 1,000 mg BID	• **Body weight, BMI, abdominal girth significantly decreased** • HOMA-IR, OGTT 2 h insulin significantly better • No effect on lipid profiles	• Menstrual periods, mFG score and Rosenfield score not changed • Testosterone, LH, LH/FSH no effect • SHBG raised (no difference between groups)
Liu *et al.* 2017 (55)	Single center, randomized, prospective, open label, 24-week, with 176 participants	Women with PCOS	Exenatide 10 μg BID for 12 weeks, then metformin 1,000 mg BID for 12 weeks	Metformin 1,000 mg BID for 24 weeks	• Body weight, BMI, WC, HOMA-IR significantly decreased • Android fat mass % and total fat mass % significantly decreased • No effect on lipid profiles	• Testosterone decreased (not significantly in both), SHBG significantly raised (no difference between groups) • Menstrual frequency ratio significantly increased • Natural pregnancy rate significantly better
Li *et al.* 2022 (62)	Follow-up of 160 participants of RCT (55)	Women with PCOS	Exenatide 10 μg BID for 12 weeks, then metformin 1,000 mg BID for 12 weeks and more (stop if pregnancy occurred)	Metformin 1,000 mg BID for 24 weeks and more (stop if pregnancy occurred)	-	• **Spontaneous pregnancy rate at 24 weeks significantly better** • Total pregnancy rate at 64 weeks (spontaneous and with assisted reproductive technology treatment) not changed • Pregnancy outcomes (miscarriage, live birth and preterm delivery, gestational diabetes and hypertension) not changed
Zheng *et al.* 2019 (54)	Single center, randomized, prospective, 12 weeks with 332 participants	Women with PCOS (182) and healthy women (150)	Exenatide 10 μg BID (only for PCOS women)	Metformin 1,000 mg BID (only for PCOS women)	• **ZAG level significantly increased (no difference between group)** • Body weight, BMI, abdominal girth significantly decreased, HOMA-IR significantly decreased • No effect on lipid profiles	• Testosterone, LH, LH/FSH no effect • SHBG raised (no difference between groups)
Tao *et al.* 2021 (61)	Single center, randomized, controlled, parallel-group, prospective, open-label, 12 weeks with 183 participants	Women with PCOS and prediabetes	Exenatide 10 μg BID	Metformin 1,000 mg BID	• **Remission rate of prediabetes significantly higher in exenatide and combination group**	-
				Combination of exenatide plus metformin		

Primary outcomes of the trials are shown in bold. Significant differences are specified in the table. ‘No difference between groups’ is stated when effects are seen in both groups but not significantly different.

Abbreviations: PCOS, polycystic ovary syndrome; sc, subcutaneous; BID, bis in die; BMI, body mass index; HOMA-IR, homeostatic model assessment for insulin resistance; SHBG, sex hormone-binding globulin; QW, every week; TID, ter in die; FPG, fasting plasma glucose; OGTT, oral glucose tolerance test; mFG, modified Ferriman–Gallwey; LH, luteinizing hormone; FSH, follicle-stimulating hormone; RCT, randomized controlled trial; ZAG, zinc-α2-glycoprotein; WC, waist circumference.

Most of them demonstrated significant weight loss, reduction in BMI and WC after treatment with exenatide compared to metformin (64, 65, 66) or with a combination treatment of metformin plus exenatide (67, 68). In addition to lowering hyperinsulinemia, exenatide treatment was found to be superior to metformin in enhancing insulin sensitivity (HOMA-IR, FGP, oral glucose tolerance test (OGTT) 2 h glucose, OGTT 2 h insulin) in some studies (65, 66, 67), while others reported no significant distinctions between the two treatments (68). The effectiveness of once-weekly exenatide has also been compared to dapagliflozin, metformin and phentermine/topiramate in a 24-week trial (69). Combining exenatide and dapagliflozin resulted in the greatest weight loss. Despite similar reductions in BMI and WC with phentermine/topiramate, only the combined treatment and single exenatide resulted in a significant improvement in glucose regulation, including mean blood glucose, insulin secretion and sensitivity index.

In 2019, a study examined the concentration of zinc-α2-glycoprotein (ZAG) in women with PCOS, its correlation with endocrine and metabolic indicators of women with PCOS, and the variations in ZAG levels after exenatide or metformin treatment (64). ZAG is a soluble protein produced and secreted by the subcutaneous and VAT. Its serum levels are lower in women with PCOS compared to insulin-sensitive women, and it may be a cytokine linked to insulin resistance in women with PCOS (70). The study’s findings revealed that ZAG levels were significantly decreased in women with PCOS, overweight/obese women, and those with elevated blood glucose levels compared to healthy women. A 12-week treatment with exenatide or metformin improved the level of ZAG (64).

In 2021, exenatide, metformin or a combination of the two was evaluated for prediabetes, a condition affecting up to 40% of women with PCOS. Compared to metformin alone, exenatide or a combination of the two achieved a higher rate of remission of prediabetes in women with PCOS by improving post-meal insulin secretion (71). These findings could be very important, as up to 70% of women with PCOS and prediabetes are estimated to develop type 2 diabetes at some point in their lives.

On endocrine outcomes, Elkind Hirsch *et al.* first demonstrated in 2008 that combining exenatide and metformin treatment for 24 weeks was superior in improving menstrual frequency and ovulation rate compared to monotherapy (using either metformin or exenatide alone) (68). In 2021, a study was carried out in China on 176 young women with PCOS who were trying to conceive. The study found that those who were treated with exenatide had a higher pregnancy rate (43.60%) at 24 weeks compared to those who only received metformin (18.70%) after a 12-week treatment period (65). However, the long-term (64 weeks) follow-up of 160 women from this previous study did not reveal any differences between the groups in terms of the total pregnancy rate (spontaneous or with assisted reproductive technology treatment) and pregnancy outcomes (miscarriage, live birth, preterm delivery, gestational diabetes and hypertension) for women who successfully conceived (72). The authors of these studies concluded that significant weight loss led to improvements in metabolic outcomes, such as insulin resistance and inflammatory markers, as well as in endocrine outcomes, such as menstrual cycle ratio and spontaneous pregnancy rate. However, Zheng *et al.* trial evaluated exenatide treatment using the same study design (12 weeks) to compare with metformin in young women with PCOS but was unable to confirm this hypothesis (66). Despite its positive impact on weight reduction, there were no significant changes in menstrual cycles, modified Ferriman–Gallwey (mFG) score (hirsutism) and Rosenfield score (acne) following a 12-week period of therapy with exenatide or metformin.

### Semaglutide in PCOS

In 2023, treatment with semaglutide at low doses (0.5 mg weekly) significantly reduced body weight in almost 80% of women with PCOS who were unresponsive to a previous lifestyle plan (73). This was associated with a significant improvement in basal glucose, insulin resistance (calculated by HOMA-IR) and in menstrual cycles. In 2024, an observational study aimed to evaluate metabolic and endocrine outcomes in 25 women with PCOS and obesity who continued treatment with metformin 2 years after discontinuation of a short-term intervention (16 weeks) with semaglutide (up to 1 mg weekly) as an add-on to metformin therapy (74). Overall, during the semaglutide treatment phase, women lost a significant amount of body weight and regained about one-third of prior weight loss, still resulting in a statistically significant net weight loss from the beginning to 2 years after semaglutide cessation. However, other metabolic and endocrine effects observed did not remain.

### Dulaglutide in PCOS

A recent study examined the impact of dulaglutide and a calorie-restricted diet (CRD) on VAT and metabolic profiles in women with PCOS. When compared to CRD alone, the addition of dulaglutide to CRD resulted in a 7% weight loss in a significantly shorter period of time, but did not show a notable difference in VAT reduction between the two groups (75). Positive benefits were reported in the dulaglutide group, showing a reduction in glycated hemoglobin A1c (HbA1c) and postprandial plasma glucose levels. Endocrine outcomes (menstrual frequency and hormonal markers) did not differ significantly. The authors emphasize the importance of dietary intervention as the initial treatment for women affected by PCOS. Improving insulin resistance, oligo-anovulation and hyperandrogenism requires a focus on enhancing adipokines and inflammatory markers through weight and visceral fat reduction.

### Beinaglutide in PCOS

In 2023, Wen *et al.* conducted a randomized study in China to compare the effects of low-dose beinaglutide combined with metformin to metformin alone on obesity indices in women with PCOS. Sixty-four women were recruited to receive either beinaglutide and metformin or metformin alone for a period of 12 weeks. The combination treatment group showed significant improvement in obesity outcomes, including body weight, BMI, WC and waist-to-height ratio (WHtR). In terms of hormones, the beinaglutide group displayed a significant drop in total testosterone levels, while antral follicular count, ovarian volume, mFG (hirsutism) and LH/FSH levels remained constant in both groups (76). Metabolic and endocrine outcomes of semaglutide, dulaglutide and beinaglutide in PCOS are presented in Table 3.

**Table 3:** Metabolic and endocrine outcomes of semaglutide, dulaglutide and beinaglutide in PCOS.

Reference	Trial characteristics	Metabolic characteristics	Intervention group	Comparative group	Metabolic outcomes	Endocrine outcomes
Carmina *et al.* 2023 (63)	Single center, prospective, open-label, 97 participants (27 PCOS patients and 65 normal ovulatory controls)	Women with PCOS and normal control	Semaglutide, 0.5 mg subcutaneously weekly for 3 months (27)	No intervention (65)	• **BMI, body weight significantly decreased** • Fasting glucose, insulin and HOMA-IR significantly decreased	-
Jensterle *et al.* 2024 (64)	Observational study, with 25 participants, 16 weeks treatment phase, then 2 years off treatment extension phase	Women with PCOS	Treatment phase: Semaglutide up to 1 mg weekly, with metformin 2,000 mg/day Off treatment extension phase: Metformin 2,000 mg/day	-	Treatment phase: • Body weight and BMI significantly decreased • Total, LDL cholesterol, HDL, fasting plasma glucose and glucose on 120 min of OGTT significantly improved Off treatment extension phase: • **Body weight and BMI significantly decreased** • Total, LDL cholesterol, HDL, fasting plasma glucose and glucose on 120 min of OGTT not changed	Treatment phase: • Free testosterone and FAI significantly decreased • LH, FSH, SHBG, DHEA not changed Off treatment extension phase: • Free testosterone, FAI, LH, FSH, SHBG, DHEA not changed
Zhang *et al.* 2023 (65)	Single center, randomized, controlled, prospective, open-label, with 68 participants (duration to achieve 7% weight loss)	Women with PCOS	Dulaglutide 1.5 mg weekly plus CRD	Calorie restricted diet alone	• **Reduction on VAT (no difference between group)**	• Improvement in menstrual frequency (no difference between group) • Testosterone decreased (no difference) • No significant change in LH, FSH, SHBG, DHEAS
Wen *et al.* 2023 (66)	Single center, randomized, prospective, open label, 12-week, pilot with 64 participants	Women with PCOS	Beinaglutide 0.1–0.2 mg TID with metformin 850 mg BID	Metformin 850 mg BID alone	• BMI, WC and WHtR significantly reduced • Fasting insulin reduced	• Testosterone level significantly reduced • LH, FSH, LH/FSH not changed • Antral follicular, ovarian volume not changed • Hirsutism (mFG score) not changed

Primary outcomes of the trials are shown in bold. Significant differences are specified in the table. ‘No difference between groups’ is stated when effects are seen in both groups but not significantly different.

Abbreviations: PCOS, polycystic ovary syndrome; BMI, body mass index; LDL, low-density lipoproteins; HDL, high-density lipoproteins; OGTT, oral glucose tolerance test; FAI, free androgen index; LH, luteinizing hormone; FSH, follicle-stimulating hormone; SHBG, sex hormone-binding globulin; DHEA, dehydroepiandrosterone; DHEAS, dehydroepiandrosterone sulfate; TID, ter in die; BID, bis in die; WC, waist circumference; WHtR, waist-to-height ratio; mFG, modified Ferriman–Gallwey; VAT, visceral adipose tissue; CRD, calorie-restricted diet.

### Dual and triple incretin-based co-agonists

The development of new molecules, such as incretin multiagonists, holds great promise in the management of various metabolic diseases, including diabetes, obesity and PCOS. Various combinations of multiagonists have already been evaluated in preclinical studies or clinical trials, such as dual agonists like GLP-1/GIP (gastric inhibitory polypeptide) or triple agonists like GLP-1/GIP/glucagon receptor. GIP receptors are expressed in the central nervous system, white adipose tissue and bone. Activation of the GIP receptor leads to an anorexigenic effect in the central nervous system and an improvement in function and anti-inflammatory effects in white adipose tissue, thus potentially exerting a synergistic and beneficial effect when combined with GLP-1 receptor activation. Tirzepatide is a dual GLP-1/GIP receptor agonist already available for the management of individuals with type 2 diabetes or overweight/obesity (77). However, data on its efficacy in women with PCOS are lacking. Due to its highly beneficial effects on weight loss and insulin sensitivity, this molecule represents a promising future therapeutic option for women with PCOS.

Retatrutide, a triple GLP-1/GIP/glucagon receptor agonist, could also represent a future therapeutic option for PCOS, given its very encouraging results in trials primarily focused on obesity and diabetes. However, future data specific to PCOS are still needed (78).

### Position of GLP-1 RAs in the management of PCOS

Regarding the position of GLP-1 RAs in the management of PCOS, their use has been incorporated into the 2023 International Evidence-based Guideline for the Assessment and Management of PCOS (5).

It is indicated that GLP-1 RAs may be considered, alongside active lifestyle interventions, for managing women with PCOS following general population guidelines. It is recommended that women of reproductive age using GLP-1 RAs should be on effective contraception during therapy and observe a washout period before attempting to conceive to minimize potential risks. Shared decision-making about GLP-1 RAs use with women with PCOS should consider the lack of long-term safety data, the potential side effects, and the likelihood of requiring long-term therapy due to the high risk of weight regain after discontinuation. The use of GLP-1 RAs during early pregnancy remains understudied, with limited data available on their safety in this context. However, findings from large patient registries involving women exposed to GLP-1 RAs in early pregnancy for the treatment of type 2 diabetes or overweight/obesity are reassuring, suggesting no significant increase in adverse outcomes (79). GLP-1 RAs could also play a role in future recommendations for the postmenopausal management of women with PCOS. This stage is often associated with weight gain and an increased cardiovascular risk, particularly in women with PCOS. As GLP-1 RAs have demonstrated cardiovascular protective effects in large randomized trials involving individuals with overweight or obesity, it is possible that these benefits could be especially advantageous for postmenopausal women with PCOS, a group at heightened risk during this period (80). However, this potential benefit will need to be confirmed through dedicated studies addressing this specific question.

## Conclusion

PCOS is a prevalent condition that remains challenging to treat, as current therapeutic options often have limited effectiveness. A pharmacological approach specifically targeting the metabolic dysfunction associated with PCOS is essential, and the therapeutic class of GLP-1 RA shows promising potential.

Current data generally support the benefits of GLP-1 RAs on several pathological aspects of PCOS, including the improvement of menstrual cycles, reduction of testosterone levels, and enhancement of glucose homeostasis. However, many available studies have limited sample sizes and follow-up durations, which restrict the overall strength of the evidence, despite a number of them being randomized. Therefore, long-term studies with larger cohorts that also evaluate other PCOS-related aspects, such as psychological disorders, are essential. A deeper understanding of the pathogenesis of PCOS is essential, along with more mechanistic data to explain the potential benefits of GLP-1 RAs for this condition. The emergence of new molecules based on incretins, such as dual GLP-1/GIP receptor agonists like tirzepatide, paves the way for future studies on this class of drugs, which are even more metabolically effective.

Identifying the clinical and biological characteristics of women with PCOS who are likely to benefit most from GLP-1 RAs therapy will be valuable in integrating this class of treatment into future recommendations for managing women with PCOS.

## Declaration of interest

The authors declare that there is no conflict of interest that could be perceived as prejudicing the impartiality of this review.

## Funding

This work did not receive any specific grant from any funding agency in the public, commercial or not-for-profit sector.
